# Health 4.0 in the medical sector: a narrative review

**DOI:** 10.1590/1806-9282.20231149

**Published:** 2024-03-15

**Authors:** Antônio Cruz, Eduardo Mário Dias, Maria Lídia Dias Scoton, Braulio Henrique Magnani Branco

**Affiliations:** 1 Universidade de Sao Paulo, Institute of Radiology, Clinical Hospital, Faculty of Medicine – São Paulo (SP), Brazil.; 2 Universidade de Sao Paulo, Polytechnic School, Department of Electrical Energy Engineering and Automation – São Paulo (SP), Brazil.; 3 Universidade de Sao Paulo, Electrical Automation in Industrial Systems Group – São Paulo (SP), Brazil.; 4 Universidade Cesumar, Health Promotion Department – Maringá (PR), Brazil.

## INTRODUCTION

The introduction of technological resources in health and medicine has led to numerous innovations^1,2^. However, there is still a lack of feedback and decisive actions to deliver services and products that reduce costs and improve evaluation, diagnosis, and medical treatment^3,4^. "Health 4.0" is a concept that encompasses the use of technologies such as Big Data, Internet of Things (IoT), cloud computing, and artificial intelligence (AI) to improve health care. These technologies can potentially improve the quality of care, reduce costs, and make health care more accessible^5^.

However, challenges must still be overcome before "Health 4.0" can be fully realized^5-8^. These challenges include the promotion of health literacy, adherence to the use of technologies, and organizing infrastructure for optimal and real-time conduction of the indicators^9-11^. This study aimed to discuss the challenges and possibilities of "Health 4.0" in the medical sector regarding information and knowledge management, efficiency and effectiveness of the service, and the current level of evidence.

## METHODS

The SANRA guideline^12^ was used to organize this narrative review. Articles indexed in the following databases were used: Latin American and Caribbean Literature in Health Sciences (LILACS), Scientific Electronic Library Online (SciELO), PubMed, and Web of Knowledge, with the following filters: (1) research published in the past 10 years (October 2012 to October 2022) and (2) systematic reviews and/or meta-analyses. The following indexing terms or descriptors in Portuguese and English were used: "health 4.0" and "big data" or "internet of things" or "cloud computing" or "artificial intelligence." A total of 23 articles on the proposed theme were included in the final analyses.

## RESULTS AND DISCUSSION

The results of this study are divided into three sessions, including "Health 4.0": (1) information and knowledge management; (2) efficiency and effectiveness of care; and (3) current level of evidence.

### Information and knowledge management in "Health 4.0"

The use of devices in 4.0 health has generated data that need to be analyzed to turn it into information that guides evidence-based practice^13^. Medical companies and professionals must establish strategies to manage these data and create mechanisms to explore the information collected^14^. One strategy is to use software that provides health professionals with specific guidelines or recommendations to assist in their diagnosis, disease management, and treatment.

This software, called Medical Decision Support Systems, can reduce diagnostic time and improve the quality of care for patients^15^. Wearable devices that feature Internet-based technologies, which have been related to monitoring the level of stress, amount, and quality of sleep, asthma, chronic obstructive pulmonary disease, cardiovascular diseases, diabetes and nutrition, aspects related to gait and falls, neurological diseases, recognition of physical activity, and rehabilitation, among other functions^16^.

The data processing systems that reproduce human cognitive functions’ speed and ability to relate and analyze information exponentially have been discussed in the literature before the economic impacts on health care^17^. Cozzoli et al.^14^ discussed that big data analyses are considered a milestone for managing studies applied to health organizations, although scientific research lacks investigations regarding the standardization and integration of devices.

The increase in the potential for big data is associated with continuing medical education, based on (1) transformation of data related to learning with medical systems; (2) intelligence in health based on learning about innovation in health and forecasting processes; (3) data collection to understand the patient's profile; and (4) learning based on clinical decision-making in health^18^. The collected data can boost learning and revolutionize the medical industry since they store up-to-date knowledge from innovative research^18^. Medical companies that acquire medical technologies, hardware, and software must also invest in continuing education and research to make informed decisions about diagnoses, treatments, medication selection, and follow-up^19^.

The conduction of randomized clinical trials is fundamental for advancing processes related to Health 4.0, such as the development of artificial technologies, big data, cloud, cybersecurity, telemedicine, and wearable devices, to improve global digital health strategies^20^. It is concluded that processes linked to Health 4.0 need to be tested on a large scale in health centers to improve the systems and the services that will be provided.

### Efficiency and effectiveness of care

The literature has discussed the economic evaluation, impact of technologies, and process management of Health 4.0. Voets et al.^21^ noted that the economic evaluation of AI is limited to financial costs, and there is a lack of short-, medium-, and long-term evaluations of possible impacts on health. Pinto de Paula Filho and Lamy^22^ pointed out that there will be no real progress in the development of Health 4.0 if medical companies do not understand the impacts of these technologies on companies and patient care.

### The current level of evidence of "Health 4.0"

The DXplain software is used to compile medical information to make possible diagnoses from laboratory data, history, and symptoms, generating a list in descending order of importance also indicating further investigations, and the HELP system, which is an integrated performance system with a computerized medical record system, which contains patient information^23^. As the doctor enters patient data, the system can make reminders and alerts, interpret data, and diagnose diseases^23^.

DXplain has a knowledge base that includes more than 2,400 diseases and more than 5,000 clinical findings in medicine^15^. The PathOS software was developed to support rapid clinical diagnosis needs; this software has proven robust after 2 years of use at the Peter MacCallum Cancer Center for analysis, genetic test reporting, and curation for cancer patients^24^. Esteva et al.^25^, using a set of 14,000 images already diagnosed by dermatologists, asked the system to recognize three types of lesions: benign, malignant, and noncancerous growths. The percentage of correct answers for the AI system was 72%, and for dermatologists, it was 66%^25^.

The technology can be applied in other specialties if the image is adapted^24^. Bhalodiya, Keung, and Arvanitis^26^ observed promising results of AI in identifying tumors via magnetic resonance. Nonetheless, the authors suggest that the algorithms must be improved. Similar responses were identified by Li et al.^27^, who argued that AI algorithms require improvements to diagnose non-alcoholic fatty liver disease more assertively. Other studies have investigated highly relevant aspects of the health and quality of life of asthmatics, such as Li et al.^28^ who used a sensor to measure airborne formaldehyde levels. However, the study systematized the prototype, but so far, the next step has not yet been carried out, which will be useful to test the equipment's efficiency in monitoring formaldehyde.

In turn, Tran, Ngo, and Tong et al.^29^ developed an application for detecting falls based on machine learning, in which the respective authors consider that the technology can differentiate a fall from some other joint event, such as sitting and jumping. Thus, it is considered that the two technologies presented in the studies^28,29^ have great potential but need to be tested in controlled clinical trials. During the COVID-19 pandemic, health technologies enabled the elaboration of remote diagnosis through devices, and the non-drug treatment of obesity and associated comorbidities^9^, drug treatment, and medical equipment were delivered to isolated areas^30^.

Another contribution was monitoring patients infected by the virus through devices and interconnected networks^30^. Al-Arkee et al.^31^ pointed out that applications to increase adherence to drug treatment of cardiovascular diseases seem to be effective, but it was discussed which components would be effectively essential for patients. The same authors mentioned that developing large-scale studies would be relevant for improving applications^31^. The study carried out by Nasajpour et al.^30^ to determine the role of technologies such as wearables, smartphone applications, and others that are based on IoT in the tracking and control of COVID-19 and how they act in the three main phases – early diagnosis, quarantine time, and after recovery – showed that, in all phases, the technology based on the IoT showed good and promising results^30,32^.

The same authors consider that fine adjustments should be made as more information about the virus's behavior is collected, as this is the only way to reduce the impacts of this type of disease significantly. Considering the heterogeneity of diagnoses^33^ before the dissemination of information, diagnosis, and direction of clinical conduct based on the responses of mobile technologies, more clinical, controlled, and randomized studies demand to be carried out to increase the assertiveness of diagnoses based on new technologies.

Recently, Akhtar et al.^34^ argued that new technologies have significantly influenced health services, with the beginning of the electronic medical record, a new era of digital health, and the emerging growth of techniques that aim to implement robotic surgeries and algorithms for machine learning, which can even replace the health professionals in the future. Additionally, Battineni, Hossain, and Chintalapudi^35^ also pointed out that the information collected in "biobanks" may predict possible pathological outcomes based on AI, which will probably lead to precision medicine research and guide the population's health services.

## FINAL CONSIDERATIONS

Health 4.0 has emerged as a promising field that could revolutionize health care. However, more research is needed to validate the effectiveness of these technologies and develop treatment protocols. Medical companies need to deeply understand the technologies already present in Health 4.0 to optimize their services. Integrating new technologies with professionals in this segment can develop predictive, preventive, personalized, and participatory work.

The information presented in this article is expected to guide future research concerning Health 4.0. In particular, randomized clinical trials with the testing of protocols, use of comparison groups, exponent technologies versus conventional treatment, and gold-standard measurement versus new measurement protocols, among other possibilities, are indispensable.

## References

[1] Mejtoft T, Lindahl O, Öhberg F, Pommer L, Jonzén K, Andersson BM (2022). Medtech innovation guide: an empiric model to support medical technology innovation. Health Technol.

[2] Lin MC, Kim TH, Kim WS, Hakanson I, Hussein A, Hung L (2022). Involvement of frontline clinicians in healthcare technology development: lessons learned from a ventilator project. Health Technol (Berl).

[3] Cutler RL, Fernandez-Llimos F, Frommer M, Benrimoj C, Garcia-Cardenas V (2018). Economic impact of medication non-adherence by disease groups: a systematic review. BMJ Open.

[4] Aceto G, Persico V, Pescapé A (2020). Industry 4.0 and health: internet of things, big data, and cloud computing for healthcare 4.0. J Ind Inf Integr.

[5] Vassolo RS, Mac Cawley AF, Tortorella GL, Fogliatto FS, Tlapa D, Narayanamurthy G (2021). Hospital investment decisions in healthcare 4.0 technologies: scoping review and framework for exploring challenges, trends, and research directions. J Med Internet Res.

[6] Dinh-Le C, Chuang R, Chokshi S, Mann D (2019). Wearable health technology and electronic health record integration: scoping review and future directions. JMIR Mhealth Uhealth.

[7] Akinosun AS, Polson R, Diaz-Skeete Y, Kock JH, Carragher L, Leslie S (2021). Digital technology interventions for risk factor modification in patients with cardiovascular disease: systematic review and meta-analysis. JMIR Mhealth Uhealth.

[8] Smith B, Magnani JW (2019). New technologies, new disparities: the intersection of electronic health and digital health literacy. Int J Cardiol.

[9] Silva JAD, Salles Painelli V, Santos IC, Marques DC, Oliveira FM, Oliveira LP (2022). No effect of combined tele-exercises and nutritional coaching on anthropometric, body composition or exercise capacity outcomes in overweight and obese women: a randomized clinical trial. Nutr Hosp.

[10] Safi S, Thiessen T, Schmailzl KJ (2018). Acceptance and resistance of new digital technologies in medicine: qualitative study. JMIR Res Protoc.

[11] Al-Jaroodi J, Mohamed N, Abukhousa E (2020). Health 4.0: on the way to realizing the healthcare of the future. IEEE Access.

[12] Baethge C, Goldbeck-Wood S, Mertens S (2019). SANRA-a scale for the quality assessment of narrative review articles. Res Integr Peer Rev.

[13] Lima FR, Gomes R (2020). Conceitos e tecnologias da Indústria 4.0. Rev Bras Inovação.

[14] Cozzoli N, Salvatore FP, Faccilongo N, Milone M (2022). How can big data analytics be used for healthcare organization management? Literary framework and future research from a systematic review. BMC Health Serv Res.

[15] Dantas BL, Maria G, Almeida G, Leite YM, Athayde C, Barros FM (2018). Sistemas de apoio à decisão médica: uma inovação na medicina. Rev Ciên Saúde.

[16] Kristoffersson A, Lindén M (2020). A systematic review on the use of wearable body sensors for health monitoring: a qualitative synthesis. Sensors (Basel).

[17] Vliet-Ostaptchouk JV, Nuotio ML, Slagter SN, Doiron D, Fischer K, Foco L (2014). The prevalence of metabolic syndrome and metabolically healthy obesity in Europe: a collaborative analysis of ten large cohort studies. BMC Endocr Disord.

[18] Au-Yong-Oliveira M, Pesqueira A, Sousa MJ, Dal Mas F, Soliman M (2021). The potential of big data research in healthcare for medical doctors’ learning. J Med Syst.

[19] Jayaraman PP, Forkan ARM, Morshed A, Haghighi PD, Kang YB (2019). Healthcare 4.0: a review of frontiers in digital health. WIREs Data Mining Knowl Discov.

[20] Manyazewal T, Woldeamanuel Y, Blumberg HM, Fekadu A, Marconi VC (2021). The potential use of digital health technologies in the African context: a systematic review of evidence from Ethiopia. NPJ Digit Med.

[21] Voets MM, Veltman J, Slump CH, Siesling S, Koffijberg H (2022). Systematic review of health economic evaluations focused on artificial intelligence in healthcare: the tortoise and the cheetah. Value Health.

[22] Pinto de Paula L, Lamy M (2020). A revolução digital na saúde: como a inteligência artificial e a internet das coisas tornam o cuidado mais humano, eficiente e sustentável. Cad Ibero-Americanos Direito Sanitário.

[23] Silva BR, Melo MC, Ribeiro MDA, Borges L (2013). Sistemas de apoio a decisão médica (SADM). Rev Eletrônica Sist Informação e Gestão Tecnológica.

[24] Doig KD, Fellowes A, Bell AH, Seleznev A, Ma D, Ellul J (2017). PathOS: a decision support system for reporting high throughput sequencing of cancers in clinical diagnostic laboratories. Genome Med.

[25] Esteva A, Kuprel B, Novoa RA, Ko J, Swetter SM, Blau HM (2017). Dermatologist-level classification of skin cancer with deep neural networks. Nature.

[26] Bhalodiya JM, Lim Choi Keung SN, Arvanitis TN (2022). Magnetic resonance image-based brain tumour segmentation methods: a systematic review. Digit Health.

[27] Li Y, Wang X, Zhang J, Zhang S, Jiao J (2022). Applications of artificial intelligence (AI) in researches on non-alcoholic fatty liver disease(NAFLD): a systematic review. Rev Endocr Metab Disord.

[28] Li B, Dong Q, Downen RS, Tran N, Jackson JH, Pillai D (2019). A wearable IoT aldehyde sensor for pediatric asthma research and management. Sens Actuators B Chem.

[29] Tran HA, Ngo QT, Tong V (2017). 2017 9^th^ International Conference on Knowledge and Systems Engineering (KSE).

[30] Nasajpour M, Pouriyeh S, Parizi RM, Dorodchi M, Valero M, Arabnia HR (2020). Internet of things for current COVID-19 and future pandemics: an exploratory study. J Healthc Inform Res.

[31] Arkee S, Mason J, Lane DA, Fabritz L, Chua W, Haque MS (2021). Mobile apps to improve medication adherence in cardiovascular disease: systematic review and meta-analysis. J Med Internet Res.

[32] Shamsabadi A, Pashaei Z, Karimi A, Mirzapour P, Qaderi K, Marhamati M (2022). Internet of things in the management of chronic diseases during the COVID-19 pandemic: a systematic review. Health Sci Rep.

[33] Cheong SHR, Ng YJX, Lau Y, Lau ST (2022). Wearable technology for early detection of COVID-19: a systematic scoping review. Prev Med.

[34] Akhtar N, Khan N, Qayyum S, Qureshi MI, Hishan SS (2022). Efficacy and pitfalls of digital technologies in healthcare services: a systematic review of two decades. Front Public Health.

[35] Battineni G, Hossain MA, Chintalapudi N, Amenta F (2022). A survey on the role of artificial intelligence in biobanking studies: a systematic review. Diagnostics (Basel).

